# Retraction Note: Proteomic analysis on *N, N′*-dinitrosopiperazine-mediated metastasis of nasopharyngeal carcinoma 6-10B cells

**DOI:** 10.1186/s12860-022-00419-4

**Published:** 2022-04-19

**Authors:** Yuejin Li, Na Liu, Damao Huang, Zhenlin Zhang, Zhengke Peng, Chaojun Duan, Xiaowei Tang, Gongjun Tan, Guangrong Yan, Wenhua Mei, Faqing Tang

**Affiliations:** 1 Medical Research Center and Clinical Laboratory, Zhuhai Hospital, Jinan University, Zhuhai, 519000 Guangdong China; 2 Medical Research Center and Clinical Laboratory, Xiangya Hospital, Central South University, Changsha, 410008 Hunan China; 3 Metallurgical Science and Engineering, Central South University, Changsha, 410008 People’s Republic of China; 4 Institute of Life and Health Engineering, and National Engineering and Research Center for Genetic Medicine, Jinan University, Guangzhou, 510632 China

**Retraction Note: BMC Biochem 13, 25 (2012)**

**https://doi.org/10.1186/1471-2091-13-25**

The Editors have retracted this article. After publication, concerns were raised about the origin in the images in Fig. 3b. The authors provided raw data to address these concerns, but the data contained image duplication between different groups and different repeats of the experiment. The Editors therefore no longer have confidence in the presented data.

Authors Gongjun Tan, Guangrong Yan, Wenhua Mei and Faqing Tang have not responded to any correspondence from the editor or publisher about this retraction notice. The Editor has not been able to obtain current email addresses for authors Yuejin Li, Na Liu, Damao Huang, Zhenlin Zhang, Zhengke Peng, Chaojun Duan and Xiaowei Tang.

